# Genomic Heritabilities and Correlations of 17 Traits Related to Obesity and Associated Conditions in the Japanese Population

**DOI:** 10.1534/g3.120.401242

**Published:** 2020-04-28

**Authors:** Olivier Gervais, Kazuko Ueno, Yosuke Kawai, Yuki Hitomi, Kazuharu Misawa, Shunsuke Teraguchi, Yen-Yen Wang, Katsushi Tokunaga, Masao Nagasaki

**Affiliations:** * Center for the Promotion of Interdisciplinary Education and Research,; ^†^ Center for Genomic Medicine, Graduate School of Medicine, Kyoto University, Sakyo-ku, Kyoto, Japan,; ^‡^ Tohoku Medical Megabank Organization,; ^†††^ Graduate School of Information Sciences, Tohoku University, Sendai, Miyagi, Japan Tohoku University, Sendai, Miyagi, Japan,; ^§^ Department of International Studies, College of International Relations, Nihon University, Mishima, Shizuoka, Japan,; ^**^ Genome Medical Science Project, National Center for Global Health and Medicine, Shinjuku-ku, Tokyo, Japan,; ^††^ Department of Human Genetics, The University of Tokyo, Bunkyo-ku, Tokyo, Japan,; ^‡‡^ Department of Microbiology, Hoshi University School of Pharmacy and Pharmaceutical Sciences, Shinagawa-ku, Tokyo, Japan,; ^§§^ Institute of Biomedical Science, Kansai Medical University, Hirakata, Osaka, Japan, and; ^***^ Immunology Frontier Research Center, Osaka University, Suita, Osaka, Japan

**Keywords:** Obesity, Heritability, Genetic correlation, Japanese population, Polygenic model analysis

## Abstract

Over the past few decades, obesity has become a public health issue of global concern. Even though disparities exist between human populations, *e.g.*, the higher liver fat content of the Japanese despite a lower body mass index (BMI), studies on the genetics of obesity still largely focus on populations of European descent, leading to a dearth of genetic data on non-European populations. In this context, this study aimed to establish a broad picture of the genetic attributes of the Japanese population, by examining a representative sample of 18,889 individuals participating in the Tohoku Medical Megabank Project cohort. We applied linear mixed model methods to 17 traits related to obesity and associated diseases to estimate the heritabilities explained by common genetic variants and the genetic correlations between each pair of traits. These analyses allowed us to quantify the SNP heritability of health indicators such as BMI (0.248 ± 0.032) and HDL cholesterol (0.324 ± 0.031), and to provide one of the few estimates of the SNP heritability of cystatin C in unrelated individuals (0.260 ± 0.025). We discuss potential differences between the Japanese and people of European ancestry with respect to the genetic correlations between urinary biomarkers and adiposity traits, for which large estimates were obtained. For instance, the genetic correlations between urine potassium level and the values for weight, BMI, waist circumference, and waist-to-height ratio ranged from 0.290 to 0.559, much higher than the corresponding estimates in the UK Biobank.

Historically, obesity first emerged as a public health concern in Western high-income countries (Caballero 2007). Nowadays most low- and middle-income countries are facing rapid increases in overweight and obesity prevalence (NCD Risk Factor Collaboration 2016a), and as of 2016, almost 40% of the world’s adult population is estimated to be overweight, defined as body mass index (BMI) > 25 (World Health Organization 2018). The health problems are expected to increase further given that waist circumference (WC) has been rising at each BMI level (Popkin and Slining 2013) and that increases in abdominal fat have been shown to substantially heighten the risks of health problems at a given BMI level (Després *et al.* 2008).

Despite this worrying global trend, there is a gap between the amount of research conducted on the genetics of obesity in populations of European descent and non-European populations (Hruby and Hu 2015; Akiyama *et al.* 2017; Stryjecki *et al.* 2018). Thorough research in all human populations is warranted given the wide range of diseases that are affected by obesity, such as hypertension, kidney disease and type 2 diabetes (Hall 2000; Gansevoort *et al.* 2013; NCD Risk Factor Collaboration 2016b), and because the association between obesity and comorbidities varies across genetic groups (Setiawan *et al.* 2016; Heymsfield *et al.* 2016). For instance, compared with non-Hispanic whites in the US population, Japanese men are more susceptible to fatty liver with small increases in BMI and generally have higher liver fat content despite their lower BMI (Azuma *et al.* 2009).

It is essential to investigate how these differences relate to genetics. Examining population-level characteristics such as heritability, which measures the proportion of the total phenotypic variation that is due to genetic variation, provides insights into this diversity between populations and key information about the genetic basis of complex traits. Given that phenotypic variation is strongly dependent on both environmental and genetic factors, changes in the environment lead to changes in heritability. Studying heritability is therefore critical not only because of the genetic diversity that exists between genetic groups, but also because of the environmental differences that can be found across the world’s populations, as well as within a given population over time.

In this study, we focused on the genetic characteristics of the Japanese population, which is largely understudied compared with populations of European descent despite obvious genetic and environmental differences. By examining 17 traits related to obesity and associated conditions in a dataset of 18,889 individuals from the Miyagi and Iwate Prefectures, in Northeast Japan, we aimed to clarify the genetic correlations between each pair of traits and the SNP heritability of these traits in the Japanese population. We discuss the similarities and differences with other populations, such as that of the UK Biobank cohort.

## Materials and Methods

### Study population

The 23K dataset of the larger 150K Tohoku Medical Megabank Project (TMM) Community-Based and TMM Birth and Three-Generation cohorts was used for this study (Kuriyama *et al.* 2016). TMM was launched in the aftermath of the Great East Japan Earthquake of March 11, 2011, and aims to contribute to the realization of personalized healthcare and medicine through the construction of an integrated biobank consisting of clinical information, genome and omics data, and biospecimens. TMM follows a prospective cohort design, targeting a total of 150,000 participants from the general population of the Miyagi and Iwate Prefectures (Hozawa *et al.* 2020).

Using information on the age and sex of participants, we analyzed the phenotypes of 17 traits related to obesity and associated conditions: systolic blood pressure (SBP), diastolic blood pressure (DBP), triglycerides (TG), HDL cholesterol (HDL-C), height, weight, BMI, WC, waist-to-height ratio (WHtR), urine creatinine (uCre), urine chloride (uCl), urine potassium (uK), serum cystatin C (CysC), serum creatinine (SCre), serum uric acid (SUA), blood urea nitrogen (BUN), and hemoglobin (Hb). For each trait, the number of individuals with data available is listed in Table 1. These traits were chosen because they represent a wide range of biomarkers, including cardiovascular risk factors (SBP, DBP, TG, HDL-C), adiposity traits (weight, BMI, WC, WHtR), and urinary and blood renal function biomarkers (uCre, uCl, uK, CysC, SCre, SUA, BUN); these are either used as a measure of obesity or related to diseases for which obesity status is highly relevant. In particular, renal function biomarkers are of interest given that the relationship between obesity and chronic kidney disease (CKD) is known to be complex, obesity paradoxically being both a risk factor for the onset of CKD and a predictor of greater survival in CKD patients (Rhee *et al.* 2016). Associations between Hb and metabolic syndrome have also been reported (Hashimoto *et al.* 2015).

**Table 1:** Baseline characteristics of the study population

Clinical trait (unit)	Males					Females				
	n	Mean	SD	Min.	Max.	n	Mean	SD	Min.	Max.
Age (years)	6,278	60.87	12.19	20	90	12,611	54.32	14.67	17	88
SBP (mmHg)	4,918	130.16	17.23	81	222	8,309	126.17	17.61	77	216
DBP (mmHg)	4,918	78.65	10.05	37	125	8,309	74.24	10.41	36	132
TG (mg/dl)	4,920	147.18	104.94	21	698	8,308	118.73	71.45	18	540
HDL-C (mg/dl)	4,920	57.97	16.15	22	151	8,308	66.21	16.12	22	154
ht (cm)	4,919	165.22	6.41	141.8	189	8,310	153.18	5.94	128.2	177
wt (kg)	4,919	66.22	10.18	38.8	117.9	8,310	54.44	8.96	30.0	104.3
BMI (kg/m^2^)	4,919	24.23	3.21	16.0	42.3	8,310	23.21	3.67	16.0	45.2
WC (cm)	4,907	85.08	8.63	60.0	130.5	8,291	81.48	9.51	56.7	126
WHtR	4,907	0.515	0.052	0.376	0.821	8,291	0.533	0.066	0.367	0.840
uCre (g/l)	6,157	1.09	0.63	0.07	4.73	11,432	0.80	0.55	0.02	4.01
uCl (g/l)	6,157	5.34	2.08	0.4	12	11,432	4.81	2.19	0.2	14.1
uK (g/l)	6,157	1.66	0.95	0.1	7.1	11,432	1.58	1.00	0.0	7.4
CysC (mg/l)	6,160	0.83	0.24	0.42	2.16	11,445	0.72	0.14	0.37	1.53
SCre (mg/dl)	6,161	0.82	0.34	0.37	2.58	11,445	0.59	0.12	0.22	1.27
SUA (mg/dl)	6,161	5.86	1.29	0.6	12.6	11,445	4.38	1.06	0.0	10.0
BUN (mg/dl)	6,161	16.19	4.45	6	42	11,445	14.26	4.00	3	35
Hb (g/dl)	6,154	14.80	1.21	8.0	19.8	10,700	13.20	1.05	6.9	18.0

BMI, body mass index; BUN, blood urea nitrogen; CysC, cystatin C; DBP, diastolic blood pressure; Hb, Hemoglobin; HDL-C, high-density lipoprotein cholesterol; ht, height; n, number of individuals; SBP, systolic blood pressure; SCre, serum creatinine; SD, standard deviation; SUA, serum uric acid; TG, triglycerides; uCl, urine chloride; uCre, urine creatinine; uK, urine potassium; WC, waist circumference; WHtR, waist-to-height-ratio; wt, weight.

To remove extreme phenotypes, participants with a BMI lower than 16 (‘severe thinness’ according to the World Health Organization international classification of BMI (1995)) were excluded, and in each sex group, individuals whose phenotypic value lay more than six standard deviations from the mean were also excluded.

The phenotypes were standardized by regressing the values for each trait on age in each sex group and converting the resulting residuals to z-scores (Supplementary Figure 1) (Yang *et al.* 2013). In the univariate and bivariate linear mixed models used for estimating heritabilities and genetic correlations, covariates comprised prefecture, genotyping platform, and the first ten eigenvectors (principal components) of the genotype data from principal component analysis (PCA) to adjust for population structure.

### Genotyping and quality control

The samples of the 23K dataset provided by the Tohoku Medical Megabank Organization for this study were genotyped with Japonica v1 and v2 (Toshiba, Tokyo, Japan), Human Omni 2.5 (Illumina, San Diego, CA, USA), and OmniExpressExome (Illumina) genotyping arrays (Nagasaki *et al.* 2015; Kawai *et al.* 2015), and imputed with IMPUTE2 version 2.2.2 (Howie *et al.* 2009) using a whole-genome reference panel of 2,049 Japanese individuals (2KJPN) (Kuriyama *et al.* 2016). All statistical models used took into account the effect of genotyping platform, as described below.

Only SNPs that were available on all platforms and had an info score over 0.5 in each platform were included in our analyses. The following criteria were applied in PLINK v1.90 (Purcell *et al.* 2007) for quality control purposes: individual call rate ≥ 98%, SNP call rate ≥ 98%, minor allele frequency ≥ 1%, and Hardy-Weinberg equilibrium (*P* ≥ 10^−6^). All individuals with a heterozygosity rate more than three standard deviations from the mean were removed.

Related individuals were removed based on identity-by-descent (pi-hat > 0.2), which was calculated after performing linkage disequilibrium pruning (-indep-pairwise 50 5 0.2); for each pair of individuals, the individual with the highest genotyping rate was kept. Pruning was performed again before calculation of the first ten principal components (-indep-pairwise 50 5 0.2) and to produce the final dataset (-indep-pairwise 50 5 0.7).

After the quality control and filtering steps, 18,889 individuals and 866,089 autosomal SNPs remained for the downstream analysis.

### Statistical analysis

The following univariate linear mixed model was used to estimate the genome-wide SNP heritabilities.

y=Xβ+u+e

with Var(u) = $G\sigma_{u}^{2}$ and Var(e) = $I\sigma_{e}^{2}$,

where y is a vector that represents the phenotypes (*i.e.*, the sex- and age-adjusted z-scores), β is a vector of fixed effects (including the overall mean, prefecture, genotyping platform, and the first ten principal components from PCA), u is a vector of random effects representing the genomic additive effect, and e is a vector of residual effects. X is the design matrix for the fixed effects, G is the genomic relationship matrix (GRM), and I is a unit matrix; $\sigma_{u}^{2}$ and $\sigma_{e}^{2}$ represent the genetic and residual variances, respectively.

A bivariate model was used to estimate the genetic correlations between each pair of phenotypes. The bivariate model, which is a direct extension of the above univariate model, was as follows.

$$\left(\begin{array}{c} y_{1}\\ y_{2} \end{array}\right)=\left(\begin{array}{cc} X_{1} & 0\\ 0 & X_{2} \end{array}\right)\left(\begin{array}{c} \beta_{1}\\ \beta_{2} \end{array}\right)+\left(\begin{array}{c} u_{1}\\ u_{2} \end{array}\right)+\left(\begin{array}{c} e_{1}\\ e_{2} \end{array}\right)$$

With

$$var\left[\begin{array}{c} u_{1}\\ \begin{array}{c} u_{2}\\ e_{1} \end{array}\\ e_{2} \end{array}\right]=\left[\begin{array}{ccc} g_{11}G & g_{12}G & \begin{array}{cc} 0 & 0 \end{array}\\ g_{21}G & g_{22}G & \begin{array}{cc} 0 & 0 \end{array}\\ \begin{array}{c} 0\\ 0 \end{array} & \begin{array}{c} 0\\ 0 \end{array} & \begin{array}{c} \begin{array}{cc} r_{11} & r_{12} \end{array}\\ \begin{array}{cc} r_{21} & r_{22} \end{array} \end{array} \end{array}\right],$$

where $\left[\begin{array}{cc} g_{11} & g_{12}\\ g_{21} & g_{22} \end{array}\right]$ and $\left[\begin{array}{cc} r_{11} & r_{12}\\ r_{21} & r_{22} \end{array}\right]$ are the variance-covariance matrices for the genetic effects and residual effects, respectively (*e.g.*, $g_{12}$ is the genetic covariance between traits 1 and 2), $y_{i}$ are *n* × 1 vectors of observations on the *i*-th trait (*n* being the number of individuals), $\beta_{i}$ are *p* × 1 vectors of fixed effects on the *i*-th trait (*p* being the number of levels for fixed effects), $X_{i}$ are *n* × *p* design matrices relating the $\beta_{i}$ vectors to the observation vectors $y_{i}$, $u_{i}$ are *n* × 1 vectors of random effects on the *i*-th trait, and $e_{i}$ refers to the vectors of random residual effects associated with each individual on the *i*-th trait. These methods are described in more detail elsewhere (Thompson 1973; Szwaczkowski 2003; Lee *et al.* 2012).

The GRMs were computed with the Genetic Complex Trait Analysis tool (GCTA) by using all SNPs that passed quality control. The following equation was used to calculate the genetic relationship between two individuals j and k (Yang *et al.* 2011):

$$f_{jk}=\frac{1}{N}\overset{N}{\underset{i=1}{\sum }}\frac{\left(x_{ij}-2p_{i})(x_{ik}-2p_{i}\right)}{2p_{i}(1-p_{i})},$$

where $x_{ij}$ and $x_{ik}$ are the genotypes of the *j*-th and *k*-th individuals, respectively, at the *i*-th SNP; $p_{i}$ is the frequency of the reference allele at the *i*-th SNP; and N is the total number of SNPs.

### Computer software

GCTA v1.91.4 (ibid.) was used to perform most of the computations and statistical analyses: *i.e.*, computation of GRMs, PCA, and univariate and bivariate Genomic Restricted Maximum Likelihood (GREML) analyses. The Average Information Restricted Maximum Likelihood (AI-REML) procedure was used to estimate variance components (Gilmour *et al.* 1995), and PLINK v1.90 (Purcell *et al.* 2007) was used to perform data filtering and quality control. R version 3.5.0 (Team RC 2019) was used to regress the phenotypes on age in each sex group and transform the residuals to z-scores, and the ggplot2 v3.1.0 package was used for data visualization (Wickham 2016).

### Ethics statement

This project was conducted in accordance with the Japanese National Ethical Guidelines for Human Genome/Gene Analysis (Ministry of Education, Culture, Sports, Science and Technology *et al.* 2013) and was reviewed and approved by the ethics committee of Tohoku University. All participants gave their written consent prior to study enrollment.

### Data availability

The analyses presented in this study were based on data accessed through the Tohoku Medical Megabank Organization (ToMMo) (https://www.megabank.tohoku.ac.jp/english/). To protect the privacy of the cohort participants, requests for the use of ToMMo biobank data for research projects should be made by applying directly to ToMMo, and are subject to review and approval by the Sample and Data Access Committee. Supplemental material available at figshare: https://doi.org/10.25387/g3.11295944

## Results

### Population characteristics

The main population characteristics are presented in Table 1. At baseline, the mean (± SD) age of the participants was 54.32 (± 14.67) for women and 60.87 (± 12.19) for men. BMI ranged from 16 to 45.2 kg/m^2^ (mean, 23.21 kg/m^2^ ± 3.67) in women and from 16 to 42.3 kg/m^2^ (mean, 24.23 kg/m^2^ ± 3.21) in men.

The participants were divided into four BMI categories: ‘thin’ (BMI < 18.5 kg/m^2^), ‘normal’ (18.5 ≤ BMI < 25 kg/m^2^), ‘overweight’ (25 ≤ BMI < 30 kg/m^2^), and ‘obese’ (BMI ≥ 30 kg/m^2^). Detailed information on the number of individuals in each BMI category is provided in Supplementary Table 1. To better visualize the profile of the target population, we also calculated the mean and standard deviation of each of the 17 traits in individuals grouped by BMI and WC category (Supplementary Tables 2 and 3). A large WC was classed as ≥85 cm in males and ≥90 cm in females, and a small WC was classed as <85 cm in males and <90 cm in females, following the clinical criteria applied for the diagnosis of metabolic syndrome in Japan (Ministry of Health, Labor and Welfare 2005).

### SNP heritability estimates

For each of the traits examined, we computed the SNP heritability by performing univariate model analyses (Table 2). Given that these values only account for the additive effect of common genetic variants, they are expected to be lower than the heritability estimates typically available from twin and/or family studies. Apart from uK, all of the heritability estimates were significant (*P* < 0.05), indicating that a substantial share of the phenotypic variation in these complex human traits is due to common genetic variants in the Japanese population. The highest heritability estimate (± SE) was that of height (0.536 ± 0.031), with that of the other traits ranging from 0.033 (± 0.023) to 0.324 (± 0.031).

**Table 2:** Summary of the SNP heritabilities estimated by using univariate model analysis, for all 17 traits

Clinical trait	*h*^2^	SE	*P*-value
SBP	0.089	0.031	1.54E-03
DBP	0.131	0.031	6.52E-06
TG	0.196	0.031	8.98E-12
HDL-C	0.324	0.031	1.68E-28
ht	0.536	0.031	8.09E-74
wt	0.256	0.032	2.62E-17
BMI	0.248	0.032	3.33E-16
WC	0.209	0.032	9.01E-12
WHtR	0.238	0.032	6.99E-15
uCre	0.044	0.024	3.00E-02
uCl	0.060	0.023	4.29E-03
uK	0.033	0.023	7.11E-02
CysC	0.260	0.025	1.46E-29
SCre	0.252	0.025	2.01E-26
SUA	0.301	0.024	1.99E-40
BUN	0.138	0.024	3.86E-10
Hb	0.204	0.026	5.55E-17

BMI, body mass index; BUN, blood urea nitrogen; CysC, cystatin C; DBP, diastolic blood pressure; h^2^, heritability; Hb, hemoglobin; HDL-C, high-density lipoprotein cholesterol; ht, height; SBP, systolic blood pressure; SCre, serum creatinine; SE, standard error; SUA, serum uric acid; TG, triglycerides; uCl, urine chloride; uCre, urine creatinine; uK, urine potassium; WC, waist circumference; WHtR, waist-to-height-ratio; wt, weight.

We also computed the heritabilities by using bivariate models to compare them with the estimates of the univariate analyses. Although bivariate models are theoretically more accurate given that they take into account the information on both traits to simultaneously estimate the random effects, in practice the difference in accuracy between the two models was negligible. For instance, the SNP heritability estimates (± SE) of height calculated by using bivariate models ranged from 0.535 (± 0.031) to 0.537 (± 0.031), which was similar to 0.536 ± 0.031 in the univariate model; and those of BMI ranged from 0.245 (± 0.032) to 0.255 (± 0.032), which was comparable to 0.248 (± 0.032) in the univariate model. Given the closeness of the results, the heritability estimates calculated with the bivariate model analyses are not presented in further detail.

### Phenotypic and genetic correlations

**Figure 1:**
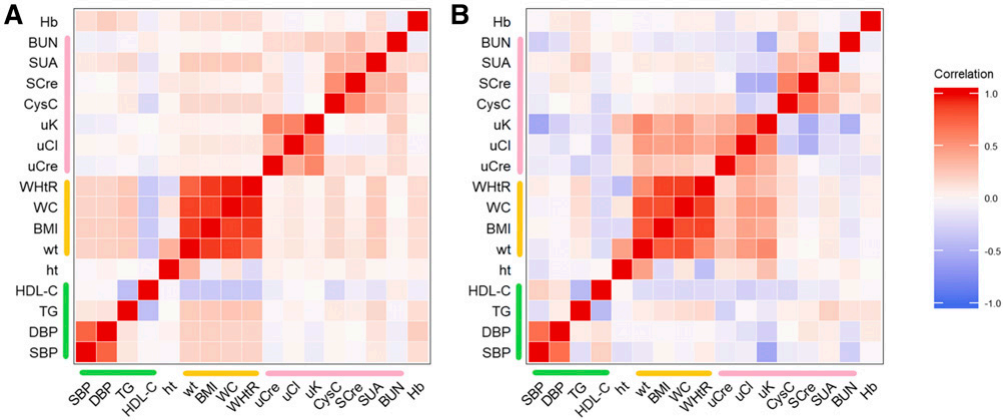
Heatmap of the phenotypic (A) and genetic (B) correlations between all 17 traits. The green, yellow, and pink lines correspond to cardiovascular risk factors, adiposity traits, and renal function biomarkers, respectively. BMI, body mass index; BUN, blood urea nitrogen; CysC, cystatin C; DBP, diastolic blood pressure; Hb, hemoglobin; HDL-C, high-density lipoprotein cholesterol; ht, height; SBP, systolic blood pressure; SCre, serum creatinine; SUA, serum uric acid; TG, triglycerides; uCl, urine chloride; uCre, urine creatinine; uK, urine potassium; WC, waist circumference; WHtR, waist-to-height-ratio; wt, weight.

Phenotypic and genetic correlations (Figure 1, Table 3) were calculated for all pairwise combinations of traits (see Supplementary Table 4 for the covariances) by using bivariate model analysis. Unsurprisingly, large correlations were found between traits representing common health markers. For instance, the genetic correlations (± SE) between BMI and WC (0.829 ± 0.028) and between BMI and WHtR (0.874 ± 0.021), which are indirect measures of adiposity, were large and significant, as were those between SCre and CysC (0.614 ± 0.045) and between TG and HDL-C (-0.429 ± 0.072). These figures are largely comparable to the corresponding phenotypic correlations, which were computed as the Pearson correlation coefficients between the sex- and age-adjusted phenotypes: 0.874 for BMI *vs.* WC, 0.890 for BMI *vs.* WHtR, 0.543 for SCre *vs.* CysC, and -0.397 for TG *vs.* HDL-C.

**Table 3:** Genetic correlations (± standard error) estimated by using bivariate model analysis (upper triangle), and phenotypic correlations (lower triangle) calculated as the Pearson correlation coefficients for the sex- and age-adjusted phenotypes, for all 17 traits.

	SBP	DBP	TG	HDL-C	ht	wt	BMI	WC	WHtR	uCre	uCl	uK	CysC	SCre	SUA	BUN	Hb
SBP		0.688 (± 0.100)**	-0.179 (± 0.175)	0.207 (± 0.135)†	-0.174 (± 0.106)*	-0.107 (± 0.156)	0.023 (± 0.151)	-0.044 (± 0.167)	0.057 (± 0.152)	-0.095 (± 0.307)	-0.075 (± 0.260)	-0.555 (± 0.389)†	-0.096 (± 0.130)	-0.082 (± 0.132)	0.051 (± 0.119)	-0.276 (± 0.176)†	-0.025 (± 0.146)
DBP	0.737		0.070 (± 0.136)	0.117 (± 0.110)	-0.026 (± 0.086)	-0.026 (± 0.126)	-0.020 (± 0.128)	-0.030 (± 0.139)	-0.014 (± 0.130)	-0.069 (± 0.253)	-0.161 (± 0.220)	-0.269 (± 0.300)	0.003 (± 0.107)	0.100 (± 0.110)	0.059 (± 0.100)	-0.169 (± 0.143)	0.082 (± 0.118)
TG	0.103	0.108		-0.429 (± 0.072)**	-0.042 (± 0.070)	0.068 (± 0.100)	0.109 (± 0.100)	0.143 (± 0.107)	0.161 (± 0.100)†	-0.262 (± 0.219)	-0.023 (± 0.177)	-0.117 (± 0.240)	0.052 (± 0.087)	0.073 (± 0.089)	0.212 (± 0.079)**	0.116 (± 0.119)	0.122 (± 0.099)
HDL-C	-0.011	-0.016	-0.397		0.007 (± 0.056)	-0.191 (± 0.075)**	-0.202 (± 0.076)**	-0.221 (± 0.081)**	-0.198 (± 0.077)**	-0.180 (± 0.166)	-0.270 (± 0.145)*	-0.203 (± 0.195)*	-0.215 (± 0.067)**	-0.087 (± 0.070)	-0.109 (± 0.064)*	0.015 (± 0.093)	-0.021 (± 0.079)
ht	-0.018	0.027	0.004	-0.027		0.454 (± 0.053)**	-0.197 (± 0.063)**	0.105 (± 0.068)†	-0.377 (± 0.062)**	0.104 (± 0.131)	0.125 (± 0.112)	0.286 (± 0.172)	-0.056 (± 0.055)	0.035 (± 0.056)	0.013 (± 0.051)	0.071 (± 0.073)	-0.002 (± 0.063)
wt	0.189	0.211	0.232	-0.312	0.363		0.784 (± 0.029)**	0.825 (± 0.028)**	0.553 (± 0.058)**	0.297 (± 0.193)†	0.477 (± 0.169)**	0.559 (± 0.266)**	0.034 (± 0.078)	-0.003 (± 0.080)	0.084 (± 0.071)	-0.070 (± 0.105)	-0.026 (± 0.090)
BMI	0.208	0.210	0.245	-0.320	-0.085	0.893		0.829 (± 0.028)**	0.874 (± 0.021)**	0.243 (± 0.193)†	0.436 (± 0.169)**	0.380 (± 0.239)*	0.078 (± 0.079)	-0.021 (± 0.081)	0.070 (± 0.072)	-0.134 (± 0.107)	-0.021 (± 0.091)
WC	0.186	0.193	0.258	-0.329	0.123	0.870	0.874		0.881 (± 0.018)**	0.228 (± 0.208)	0.482 (± 0.184)**	0.474 (± 0.269)*	0.119 (± 0.085)†	-0.078 (± 0.089)	0.105 (± 0.077)†	-0.093 (± 0.116)	0.049 (± 0.097)
WHtR	0.188	0.179	0.252	-0.315	-0.197	0.739	0.890	0.947		0.164 (± 0.193)	0.391 (± 0.170)**	0.290 (± 0.233)†	0.140 (± 0.079)*	-0.083 (± 0.083)	0.085 (± 0.073)	-0.118 (± 0.109)	0.051 (± 0.091)
uCre	-0.062	-0.044	-0.020	-0.087	0.046	0.066	0.049	0.049	0.034		0.520 (± 0.260)†	0.374 (± 0.345)	0.139 (± 0.160)	-0.015 (± 0.167)	-0.091 (± 0.152)	-0.152 (± 0.229)	-0.137 (± 0.192)
uCl	0.019	0.006	0.035	-0.021	0.016	0.098	0.096	0.086	0.079	0.388		0.575 (± 0.245)†	-0.281 (± 0.143)*	-0.481 (± 0.156)**	-0.175 (± 0.128)†	-0.137 (± 0.193)	0.062 (± 0.158)
uK	-0.090	-0.071	-0.015	-0.005	0.042	0.050	0.033	0.033	0.019	0.581	0.589		-0.185 (± 0.196)	-0.510 (± 0.260)**	-0.203 (± 0.186)	-0.501 (± 0.342)*	0.044 (± 0.210)
CysC	0.016	0.019	0.084	-0.221	0.020	0.166	0.168	0.174	0.167	0.109	-0.067	-0.005		0.614 (± 0.045)**	0.340 (± 0.057)**	0.093 (± 0.090)	0.143 (± 0.079)*
SCre	-0.021	0.002	0.061	-0.070	0.093	0.096	0.063	0.042	0.013	0.137	-0.073	0.039	0.543		0.285 (± 0.059)**	0.246 (± 0.086)**	0.046 (± 0.080)
SUA	0.080	0.108	0.167	-0.142	0.025	0.228	0.230	0.240	0.227	0.058	-0.083	0.027	0.335	0.333		-0.061 (± 0.088)	-0.015 (± 0.074)
BUN	-0.064	-0.076	-0.033	0.070	-0.014	0.017	0.026	0.004	0.009	0.149	0.151	0.217	0.190	0.311	0.156		-0.091 (± 0.104)
Hb	0.135	0.219	0.142	-0.069	0.035	0.143	0.139	0.161	0.147	0.033	-0.034	-0.001	0.051	-0.015	0.146	-0.092	

BMI, body mass index; BUN, blood urea nitrogen; CysC, cystatin C; DBP, diastolic blood pressure; Hb, Hemoglobin; HDL-C, high-density lipoprotein cholesterol; ht, height; SBP, systolic blood pressure; SCre, serum creatinine; SUA, serum uric acid; TG, triglycerides; uCl, urine chloride; uCre, urine creatinine; uK, urine potassium; WC, waist circumference; WHtR, waist-to-height-ratio; wt, weight.

^†^ P < 0.1.

* P < 0.05.

** P < 0.01.

High genetic correlations were also observed among urinary traits, *e.g.*, uK *vs.* uCl (0.575 ± 0.245). Although this is consistent with the pattern observed above, it is an interesting result given the low heritability of these traits. This finding highlights the fact that a low contribution of genetic components to phenotypic variation for a given set of traits does not necessarily imply a low genetic correlation between these traits. On the other hand, the low heritability of these traits was reflected in the higher standard error of the corresponding genetic correlation coefficients.

The phenotypic correlations were stronger between CysC and each of the adiposity traits (weight, BMI, WC, WHtR) and HDL-C than between SCre and these traits; this result supports previous research that showed that CysC is more closely associated with obesity than SCre (Ying *et al.* 2017). A similar finding was observed for genetic correlations: significant estimates were detected between CysC and WHtR and between CysC and HDL-C but not between SCre and these traits.

## Discussion

### The effect of WC on health indicators

By dividing the target population by BMI and WC group (Supplementary Tables 2 and 3), we observed that regardless of gender, in normal weight and overweight individuals, within each BMI category the subgroups with a large WC displayed significantly higher weight, WHtR, higher CysC and SUA levels, and a lower HDL-C level than those with a small WC (*P* < 0.01 in all subgroups, except SUA in overweight men for which *P* < 0.05). When examining women and men of normal weight only, the subgroups with a large WC also displayed higher DBP and higher TG and Hb levels (*P* < 0.01). More surprisingly, however, overweight men with a small WC displayed lower CysC levels than men with a normal BMI and a large WC (*P* < 0.01). These findings strongly suggest that increases in WC within each BMI category are associated with a worsening of many health risk indicators in both women and men; they are also in line with previous research indicating that abdominal fat, as measured by WC, may represent a substantial health risk factor independently of BMI, and that WC may be an effective tool for the detection of at-risk individuals, including normal weight individuals (Després *et al.* 2008).

### Heritabilities and genetic correlations of the traits in the Japanese population

The heritability estimates calculated for the Japanese population were largely comparable to those from previous research conducted in populations of European descent. For example, in a recent phenome-wide heritability analysis of the UK Biobank carried out in British (Caucasian) individuals, the heritability estimates (± SE) were 0.685 (± 0.004) for height, 0.274 (± 0.004) for BMI, 0.277 (± 0.004) for weight, 0.155 (± 0.004) for WC, 0.156 (± 0.004) for SBP, and 0.184 (± 0.004) for DBP (Ge *et al.* 2017). In another study, performed in the Lifelines Cohort (Netherlands), the heritability estimates (± SE) were 0.489 (± 0.032) for height, 0.248 (± 0.032) for BMI, 0.173 (± 0.032) for SBP, 0.170 (± 0.033) for DBP, 0.186 (± 0.034) for HDL-C, 0.191 (± 0.035) for TG, 0.190 (± 0.032) for Hb, and 0.268 (± 0.032) for SCre (Nolte *et al.* 2017). Our findings are also in line with previous estimates of the SNP heritability of CysC (0.27; 95% CI: 0.15-0.39), SUA (0.144 ± 0.039), uCre (0.065 ± 0.003), and uK (0.042 ± 0.002) (Chen *et al.* 2015; Zanetti *et al.* 2018; Nakatochi *et al.* 2019). This implies that, in spite of the genetic and environmental differences that exist between European and Japanese populations, overall the contribution of common genetic variants to phenotype variation between these groups is comparable.

A major study on BMI in a large Japanese cohort reported the discovery of dozens of novel loci, highlighting the genetic diversity between Japanese and European populations (Akiyama *et al.* 2017). This led the authors to suggest differences in the causal variants and their effect sizes as potential explanations for their findings, and to advocate for more research in diverse ethnic groups to uncover the genetic processes underlying BMI susceptibility. Although that call is certainly warranted given the well-established genetic diversity across human populations, our study of the SNP heritability of obesity-related traits indicates that the impact of these differences on phenotypic variation between Japanese and European populations is likely to be moderate. Conversely, as this example illustrates, similarity in heritability estimates does not necessarily imply similarity in terms of genetic architecture; whether the genetic architecture of each trait is similar in Japanese and European populations is an essential research topic that requires further inquiry.

Several observations deserve to be emphasized with respect to the genetic correlations (Table 3). First, although the phenotypic and genetic correlations among the three urinary traits examined (uCre, uCl, and uK) were similar in terms of sign and magnitude, sharp differences between phenotypic and genetic correlations were observed when comparing this group of urinary traits to the group of adiposity traits (weight, BMI, WC, and WHtR), as illustrated by Figure 1: the highest phenotypic correlation between any pair of traits among these two groups was below 0.1, whereas the corresponding genetic correlations ranged from 0.164 to 0.559, the highest genetic correlation being between uK and weight (*P* < 0.01).

In people of European descent, a recent study in the UK Biobank has identified a relationship between these two groups of traits, yet with much lower genetic correlations (*e.g.*, 0.104 between uK and BMI, *vs.* 0.380 in our study) (Zanetti *et al.* 2018). Given the dearth of research on the genetic architecture governing urinary biomarkers and their relationship with obesity-related traits, it is difficult to completely rule out that these discrepancies are caused by differences in methodology; however, the unmistakable differences that have been highlighted in the literature between individuals from Japan and western Europe in the relationship between adiposity, lipid biomarker data, kidney disease, and cardiovascular risk may indicate that the strength of the correlations between urinary traits and adiposity traits is specific to the Japanese (Nakamura *et al.* 2012; Wanner *et al.* 2016).

On a different note, significant genetic correlations were found between HDL-C and adiposity traits (weight, BMI, WC, WHtR), renal function biomarkers (uCl, uK, CysC, SUA), and cardiovascular risk factors (TG, SUA). This highlights the complex, intertwined relationship between these traits, as do the large negative genetic correlations between SCre and uK and between SCre and uCl (*P* < 0.01). Surprisingly, however, despite phenotypic correlations of over 0.2, we could not reproduce genetic correlations between BMI and SBP, DBP, TG, or SUA (Kanai *et al.* 2018), although we found significant genetic correlations between SUA and HDL-C and between SUA and TG.

Even though the correspondence between phenotypic and genetic correlation estimates is not a systematic result (Lynch and Walsh 1998), it has been observed previously (Vattikuti *et al.* 2012). In this study, we found correspondence between these estimates to a certain degree. For instance, the absolute value of the maximum difference between the phenotypic and genetic correlations was below 0.3 for all traits, excluding the three traits with the lowest heritabilities: uCre, uCl, and uK. Furthermore, the Pearson correlation coefficient calculated for phenotypic correlation *vs.* genetic correlation estimates for the 17 traits was high (0.767). These results therefore seem to support the view that phenotypic correlations may prove valuable in the context of linear mixed model methodology, for instance in the calculation of the preliminary estimates of the parameters used for the initialization of maximum likelihood procedures.

**Figure 2:**
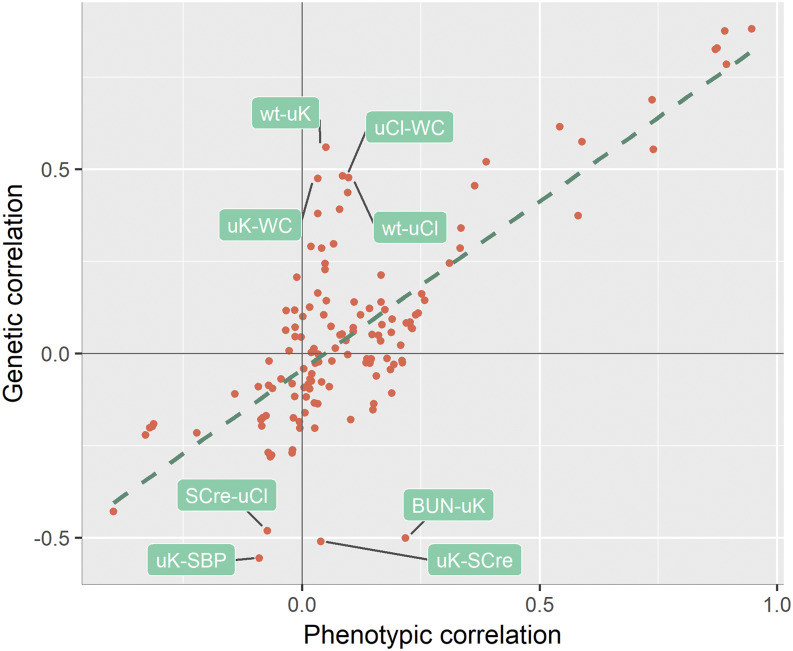
Scatter plot of the genetic and phenotypic correlations for each pair of traits and corresponding regression line. BUN, blood urea nitrogen; SBP, systolic blood pressure; SCre, serum creatinine; uCl, urine chloride; uK, urine potassium; WC, waist circumference; wt, weight.

At the same time, all three traits with low heritability, *i.e.*, the traits for which environmental factors accounted for almost all of the variation in phenotype, displayed a difference of over 0.3 between the phenotypic and genetic correlations with at least one of the other traits. Figure 2 also indicates much variation between the phenotypic and genetic correlations for the pairs of traits whose phenotypic correlation coefficients were close to zero. Although this issue needs to be examined in more depth to draw a firmer conclusion, our observation that the differences between phenotypic and genetic correlations depend on the trait pairs analyzed may partly explain why there is no consensus on the conformity of the relationship between phenotypic and genetic correlations, and leads us to recommend exercising ample caution when using phenotypic correlations as proxies for genetic correlations.

### Obesity and biomarkers for obesity-related diseases

A substantial contribution of this study is the estimation of the genetic correlations between traits associated with obesity and indicators used as health markers for obesity-related diseases. The link between obesity and obesity-related diseases is often complex, and furthering our understanding of this relationship requires undertaking a variety of analyses on a wide range of traits and in diverse populations. This complexity is well illustrated by the case of CKD: despite the known strong association between metabolic syndrome and CKD, it has been reported that traditional CKD risk factors (such as diabetes mellitus and hypertension) may be independent of kidney dysfunction in obesity, and that kidney dysfunction may appear long before these factors develop in individuals affected with metabolic syndrome (Singh and Kari 2013; Sarathy *et al.* 2016). The large genetic correlations that we found between adiposity traits and urinary biomarkers (*e.g.*, 0.436 between BMI and uCl; 0.380 between BMI and uK), as well as between SBP and uK (-0.555), provide a starting point for further research into how the genetic architecture of these biomarkers fits into the broader context of disease onset and etiology.

More broadly, these observations underscore the fact that the relationship between obesity and obesity-related diseases is not a simple cause-and-effect relationship, and compel us to revisit and better quantify the connection between the many associated traits and biomarkers, even if they typically display a somewhat low heritability and do not necessarily constitute the deciding factor in terms of disease diagnosis.

### Limitations and strengths of this study

Given that our main objective was to shed light on the genetic characteristics of the Japanese population, our findings may not be generalizable to other human populations or age groups. This is partly due to the diversity found across genetic groups, but it is also a consequence of the physiological and social differences that exist between human populations, as illustrated for instance by the differences in the WC threshold values used for the diagnosis of metabolic syndrome in Japanese and European populations. Factors such as smoking, alcohol consumption, and physical activity, as well as potential subgroup effects within the cohort, are also aspects that have not been addressed in this paper and warrant further research. When comparing our findings with the results of other studies, it is also important to keep in mind that the TMM project targets the regions affected by the Great East Japan Earthquake of March 11, 2011.

On the other hand, this study is one of only a few analyses of large-scale cohorts that focus on identifying the general genetic characteristics of the Japanese population; it includes rare and valuable information, such as one of the few estimates of the SNP heritability of cystatin C in unrelated individuals, as well as estimates of the genetic correlations between urinary biomarkers and obesity-related traits. Also, given that this study was based on high-quality clinical data, and that participants were recruited as part of the general population regardless of their medical background, it constitutes a useful point of comparison for similar studies conducted in the Japanese and other populations.

## Conclusion

By quantifying the genomic parameters of a large cohort of Japanese adults, our study provides evidence that the contribution of common SNPs to phenotype variation is significant in the Japanese for traits related to obesity and obesity-related diseases, and therefore supports the assumption that common genetic variants account for a considerable share of phenotypic variation. Our study also shows that the heritability estimates for these traits are similar in magnitude to those of populations of European descent, and suggests potential differences with respect to the genetic correlations; however, further research is needed to compare the genetic architecture of each trait in these populations. The heritabilities presented in this paper are useful in that they provide information about the proportion of phenotypic variance that could be explained by common-variant GWAS of the traits studied, and the high genetic correlations estimated for several pairs of traits with low heritabilities underscore that we must be careful not to neglect research on the genetics of biomarkers with low heritabilities.
